# Prevalence and curriculum of sexual and gender minority education in Japanese medical school and future direction

**DOI:** 10.1080/10872981.2019.1710895

**Published:** 2020-01-13

**Authors:** Yuka Yamazaki, Akiko Aoki, Junji Otaki

**Affiliations:** a Department of Medical Education, Tokyo Medical University, Tokyo, Japan; b Department of Rheumatology, Tokyo Medical University Hachioji Medical Center, Tokyo, Japan

**Keywords:** Competency, sexual and gender minority, medical education, undergraduate education, questionnaire, stigma, Japan

## Abstract

**Background**: In Japan, sexual and gender minorities (SGM) remain stigmatized, provoking hospital access barriers and health disparities from judgmental care. Japan’s Western-influenced introduction of SGM course content into medical education for future physicians addresses these disparities, although often perfunctorily and inconsistently.

**Objective**: To examine the prevalence and characteristics of medical education curriculum with respect to SGM patients, we surveyed medical schools.

**Methods**: A medical education faculty member from each of 80 Japanese medical schools received double postcards to identify relevant SGM coursework. Upon acknowledgement, 43 schools received seven-item anonymous questionnaires in March 2018. Survey results were analyzed from the perspective of three of the qualities and abilities required of a physician – Patient Care, Knowledge for Practice, and Professionalism from Japan’s Medical Core Curriculum – to develop recommendations for outcomes-based SGM curriculum through the lens of Van Melle’s medical education framework.

**Results**: The response rate was 46%, with 22 schools providing SGM lectures mostly to first- and third-year students. Obstetrics and Gynecology, Neuropsychiatry, and Introduction to Medicine lectures were the top three subjects offering SGM lectures, primarily consisting of basic knowledge of SGM and Differences in Sex Development. Several lectures addressed the health challenges of SGM. Primary reasons for not offering SGM lectures were lack of suitable instructors or no school policies.

**Conclusions**: Students can best experience the humanity of SGM patients and employ more appropriate diagnostic practices and modes of treatment with targeted curriculum to address SGM health disparities and inclusion of SGM patients in clinical practice training. To disseminate SGM education in Japanese medical schools, development of qualified instructors and policies is essential, employing currently active experts. The Van Melle *reforms* framework can guide in the development of recommended tailored learning experiences and lectures for improved and expanded SGM education, integrating appropriate coursework within current medical core curriculum structure.

## Introduction

Lesbian, Gay, Bisexual, Transgender, and Queer (LGBTQ) individuals face higher rates of obesity, substance abuse, sexually transmitted disease, and suicide than heterosexual and cisgender people [1]. In addition, they are detached from the formal healthcare system and are less likely to be insured than heterosexual and cisgender people in the United States [2]. However, Japan has universal healthcare insurance [3], providing healthcare system access to all. Nevertheless, many sexual and gender minorities (SGM) do not seek medical assistance for fear of incomprehension, discrimination, and rejection by healthcare providers [4,5].

In Japan, the stigma, discrimination, and prejudice associated with SGM hinder many from coming out to others [5–7], with some preferring to identify with heterosexual and cisgender people [7]. However, if SGM seek medical care, they often face situations in which they must come out. For example, transgender patients often must disclose to medical personnel that their gender assigned at birth is different from their gender expression, and provide the reason for this change [8]. Moreover, sexual minorities such as gay and bisexual individuals are afraid of coming out in hospitals. For example, one cross-sectional study conducted in 2014 revealed that about 53% of gay and bisexual males may have depression or mood disorders. However, the rate of consultation for psychosomatic medicine or psychiatry in the 6 months following the survey time was only 14%, implying gay and bisexual individuals may face difficulties accessing hospitals [9]. Moreover, those who have a same-sex partner like lesbian and gay individuals may face unequal treatment in hospitals. For example, because they are not lawful spouses, privacy laws prevent doctors from sharing patient medical data with sex partners, who also cannot sign consent forms for treatment and surgery [10]. At times, gay and lesbian individuals do not share the existence of partners [10]. These individuals may instead tell health providers that the same-sex partners are siblings or relatives because they are afraid of health providers being judgmental, which creates further discomfort with treatment or admission to a hospital [10].

Moreover, in Japan, providing assistance to SGM also creates stigma. Sherriff et al. reported that in Japan, the most critical prohibiting factor to preventing HIV infection is stigma associated with sexual minorities [11]. For example, stigmatization of sexual minorities historically resulted in a slow response to the epidemic of HIV compared to many Western European cultures, and currently continues to result in very low levels of financial support from Japanese local and national governments to non-governmental organizations (NGOs) conducting HIV prevention and care [11].

Recently, however, even in Japan’s harsh environment, alleviation of the stigma towards SGM has begun, owing to Western influence. For example, regarding discriminatory laws and practices as well as acts of violence against individuals based on their sexual orientation, Japan was influenced by a resolution on human rights violations of the United Nations in 2011 [12] and a US Supreme Court ruling to legalize same-sex marriage in 2015 [13]. As a result, Shibuya ward in Japan was the first to recognize same sex-marriage in 2015 [14]. In the same year, the Ministry of Education, Culture, Sports, Science and Technology (MEXT) instructed teachers to address concerns of sexual and gender minority students in junior and senior high school [15].

In addition, the *Model Core Curriculum for Medical Education in Japan AY 2016* by the Ministry of Education, Culture, Sports, Science and Technology incorporated as a competency ‘the ability to explain gender formation, sexual orientation, and ways of consideration for gender identification’ [16]. Japanese medical schools developed their own curriculum based on the *Model Core Curriculum*. Japan’s English version of the *Model Core Curriculum*, and its approach to SGM education, is presented in Table 1 in the Appendix. Although applicable to all people, the *Model Core Curriculum* also states that physicians should consider the relationship between culture, gender, and medical care [16].

**Table 1.** Explanation of Model Core Curriculum and background of introduction of sexual and gender minority education

Model Core Curriculum	‘The Model Core Curriculum for Medical Education is an abstraction of the “core”, within the respective “curriculum” formulated by each university, which should be taught in common by all universities in Japan; it is systematically organized as a “model” of what that core contains’
Background of introduction of sexual and gender minority education.	Model Core Curriculum was revised in 2016 with the goal of cultivating physicians who can serve diverse needs. The revision considered many changes occurring in international public health and healthcare systems, and is meant to cultivate physicians with practical clinical capability, who can serve the needs of the public in areas such as ethics, medical safety, team-based healthcare, the community-based integrated care system, and a healthy long-living society. This curriculum added ‘Human behaviour and psychology’ in General issues in medicine, which includes an item referring to ‘Individual differences'. Moreover, one of the objectives of ‘individual difference’ is ‘Explain gender formation, sexual orientation, and ways of consideration for gender identification'.

Medical Education Model Core Curriculum Coordination Committee and Medical Education Model Core Curriculum Expert Research Committee, 2017.

Ultimately in April 2018, universal insurance started to cover gender reassignment surgery, but not hormonal therapy [17,18]. Previously, transgender people had depended upon foreign countries to have gender reassignment surgery and import hormonal drugs privately [17,18]. However, these practices often brought about financial problems, surgical complications, or adverse drug reactions [17,19].

Medical education related to SGM is essential for LGBTQ quality of health [20]. Kelley et al. demonstrated that medical students who received instruction on sexuality and Lesbian, Gay, Bisexual, Transgender, and Questioning/Queer (LGBTQ) health felt better-equipped and more comfortable treating patients who identified as LGBTQ, and increasingly understood the clinical relevance of sexual orientation and gender identity [21]. In practice, more systematic training of healthcare professionals is needed for skilled treatment of SGM as patients. At the same time, understanding is critical as to how social and economic determinants, biases, inequalities, and blind spots shape health and illness long before doctors and patients enter examination rooms [20].

Particularly in Japan, incorporating a curriculum on SGM issues in medical schools is crucial to alleviating stigma among healthcare providers. However, even in Western countries, incorporating healthcare for SGM patients into the curriculum is not an easy task, and a variety of efforts have been made to overcome this challenge [22]. At present, only two Japanese medical schools shared outlines of their SGM lectures [23,24]. Thus, the number of medical schools providing SGM curriculum, or the type of lectures being given, is not known. In the Japanese medical educational system, applicants can take an entrance examination during their final year in high school [25]. After entry, students spend 6 years studying liberal arts, basic sciences, clinical medicine, and bedside medicine [25]. Generally, first-year medical student course content focuses on liberal arts [26], and second-year to mid-third-year content, basic sciences. Mid-third-year and fourth-year students develop knowledge and skills in clinical medicine. Fourth-year students begin bedside medicine [27]. After graduation, a 2-year general residency has been required of all medical graduates [28].

In Western countries, several studies have examined medical students’ knowledge of attitudes and clinical skills with respect to SGM patients using questionnaire surveys [29–31]. Japanese medical education has not yet attained the level wherein student knowledge of the clinical needs of SGM patients is examined. Therefore, as a first step, we conducted a survey by mail to examine the prevalence of SGM education, its structure, how lectures complied with competencies, barriers for dissemination of this education, and recommendations for implementation.

## Methods

### Selection of subjects

*First*, we selected one faculty member from each medical school using a list of faculty members from a medical education organization representing faculties in 80 Japanese medical schools. Those selected were full-time and highly ranked faculty members in each medical education department. About 78.7% of selected faculties were professors and 87.5% were males according to their first names.

*Second*, we sent the 80 faculty members double postcards in February 2018, requesting them to furnish referrals to key persons who could provide accurate information about SGM lectures in their medical schools. After sending the postcards, we also sent emails through mailing lists of medical education organizations encouraging the addressees to take the survey and return the cards to us. In addition, we resent the reminder emails through the same mailing list right before the deadline.

### Subjects

A total of 47 medical schools returned the double postcards, with two schools sending us emails stating that they would not reply to the postcards. Four of the 47 medical schools responded that contact names were not given because two of those schools did not have SGM lectures, one school did not have a contact person, and one school recommended that an author of the present study fill out the survey as the contact. Thus, a total of 43 schools were considered as study targets, of which 33 faculty members belonged to medical education departments and 10 to other departments.

### Data collection

The anonymous questionnaires were distributed to the subjects’ offices by mail, using the address written on the medical schools’ website, and were returned by mail. Upon receipt, completed questionnaires were immediately labelled with an identification number, and then processed in a delinked, anonymous manner. The study data were collected in March 2018.

### Ethical approval

Not applicable. The Clinical Research Support Center of Tokyo Medical University waived the need for IRB approval as this study focused on lectures provided by departments, and not on information regarding individual students.

### Questionnaire contents

The seven-item questionnaire (Appendix) requested information regarding the following items: (1) existence of SGM classes or workshops, (2) whether these classes or workshops were required or elective, (3) the class level of students to whom these classes or workshops were offered, (4) details about these classes or workshops including the time allocation, class title, and content summary, (5) reason(s) for not offering any SGM classes or workshops, (6) any future plans to offer SGM classes or workshops, and (7) ideal fields or departments to offer SGM classes and workshops.

### Survey data analysis within core curriculum and Van Melle framework

We examined the prevalence and characteristics of SGM curriculum in Japan’s medical training reported in survey results. In our analysis, we considered our qualitative data in the context of medical core curriculum within a medical training framework to develop recommendations for strategies that integrate SGM curriculum into Japan’s medical education. *Model Core Curriculum for Medical Education in Japan* provides professional and clinical learning standards, a rough summary of expected progression, and an emphasis on societal impact [16].

In education, a framework is used as a roadmap, identifying teaching responsibilities demonstrated by empirical studies and theoretical research to promote student learning and outcomes [32]. With the recognition that a significant shift in behaviours would be warranted for SGM curriculum to be effectively implemented in Japan, we structured recommendations consistent with the competency-based Van Melle *reforms* framework, advocating transformative change to reflect the scale of the needed outcomes [33]. The Van Melle framework focuses on the learner, stressing curricular outcomes, and favouring the reinforcement and strengthening of abilities over standard time-based training [33]. Competency-Based Medical Education (CBME) leverages outcome competencies, sequenced progression, tailored learning experiences, competency-focused instruction, and programmatic assessment to achieve stated competencies and desired outcomes [33]. In the *reforms* model, a checklist-based approach is discouraged due to the dynamic nature of evolving elements [33,34]. Thus, we developed fundamental recommendations for enhancing the delivery of SGM education in Japan’s medical curricula with the aid of the Van Melle framework [33].

Japan’s English version of the *Model Core Curriculum for Medical Education*, under *Human Behavior and Psychology: Individual Differences*, establishes one competency directly related to SGM curriculum, ‘the ability to explain gender formation, sexual orientation, and ways of consideration for gender identification’ [16]. However, as a singular aim, this competency is overly broad, so we structured our competencies based on three of the qualities and abilities required of a physician classifications in the Model Core Curriculum for Medical Education in Japan AY 2016 and designated them as our primary competency classifications: [1] Patient Care, [2] Knowledge for Practice (Practical Skills in model core), and [3] Professionalism (as further described in ‘Implementing Curricular and Institutional Climate Changes to Improve Health Care for Individuals Who Are LGBT, Gender Nonconforming, or Born with DSD’ [16,35]). These three classifications were selected because: [1] they reflect a subset of the qualities and abilities of physicians in the Japanese core curriculum, and provide structural parallel for the organization of core competencies, [2] our education regarding SGM had not been applied to practical settings, and therefore needed a classification structure from existing core models. We selected competencies related to knowledge and attitudes that physicians should acquire when they see SGM patients. Competencies were compiled from observations extracted from questionnaire results and viewed through the lens of *Japan’s Model Core Curriculum in Medical Education* [16] and the Van Melle Framework [33].

## Results

The response rate to the survey was 46.3% when we applied all medical schools (80) as the denominator. The results of this survey are shown in Tables 2–5.

**Table 2.** Prevalence of sexual and gender minority education – including all 80 of Japan’s medical schools into denominator

Sexual and gender minority education(class or workshop)	N	%
Yes	22	27.5
No	14	70.9
Unknown	1	1.6

**Table 3.** Summary of sexual and gender minority educational coursework (N = 22)

Type of class or workshop	N	%
Required	19	86.3
Elective	2	9.1
No Answer	1	4.5
Target academic year level (multiple answers)	N	%
First	9	30.0
Second	5	16.7
Third	9	30.0
Fourth	5	16.7
Fifth	1	3.3
Sixth	1	3.3
Median time of lecture or workshop	130 (min)	
Offered subjects (multiple answers)	N	%
Obstetrics and Gynecology	4	11.4
Neuropsychiatry	4	11.4
Introduction to Medicine	4	11.4
Ethics	3	8.6
Urology	2	5.7
Pediatrics	2	5.7
Introduction to Diagnosis	1	2.9
Professionalism	1	2.9
Human Rights Education	1	2.9
Healthcare and Society	1	2.9
Physician–Patient Relationships	1	2.9
Medical Education Workshop	1	2.9
Genetic Inheritance and Genes	1	2.9
English	1	2.9
Behavioral Science	1	2.9
Behavioral Therapy	1	2.9
Integrated Lecture	1	2.9
Freshman Seminar	1	2.9
Psychology	1	2.9
Male Organs and Retroperitoneal Diseases	1	2.9
Sociology	1	2.9
Others	1	2.9

**Table 4.** Extraction of sexual and gender minority course contents from 22 medical schools

Target Academic Year	Offered subjects	Course Outline
2	Professionalism	Introduction to sexual and gender minority
4	Professionalism	Treatments for Gender Identity Disorder
1	Freshman Seminar (Educational subject in first year)	As a group work, freshmen at medical college discussed the news which one women university was considering the enrollment of a transgender student (male to female)
4	Pediatrics and Obstetrics and Gynecology	Difference of Sex Development (DSD)
1	Ethics	Discussion about the case which lesbian women have a baby with artificial semination
5	Introduction to Medicine	Sexuality and Gender Diversity, A lecture conducted by those who identify with these groups, Contemplation about LGBTQ related issues
2	Medical Ethics	Concept about LGBTQ
4	Healthcare and Society	History, social problems, and discrimination about LGBTQ
3	Physician-Patient Relationships	Sexual and gender minority
3	Obstetrics and Gynecology	Indeterminate sex, Turner syndrome, Klinefelter’s syndrome, Prader disease, Swyer syndrome, androgen insensitivity syndrome, congenital adrenocortical hyperplasia, (Mayer–Rokitansky–Küster–Hauser) MRKH syndrome, congenital anomaly of uterus
3	Medical Education Workshop	‘To understand biological characteristics, sexual identity, sexual orientation, and sexual expression through the experiences of those who identify with these groups,’ and ‘To discuss the roles of healthcare professionals’
1	Psychiatry	anxiety and difficulties that persons with gender Identity Disorder (GID) and gender dysphoria are likely to experience
3	Introduction to Medicine (Psychiatry)	Medical interventions for transgender patients: hormone therapy and gender-affirming surgeries (what does determine sex?)

**Table 5.** Reasons for schools not offering sexual and gender minority educational coursework (N = 15:14 schools not offering lectures and 1 school with unknown status)

Reasons for not offering sexual and gender minority education (multiple answers)	N	%
No suitable instructors	8	27.5
No policy in medical schools	6	20.7
Not having concrete ideas about what to do	4	13.8
Curriculum is too tight to offer a class	3	10.3
Unable to find standard teaching materials	3	10.3
Currently no need has been identified	2	6.9
Others	3	10.3
Future plans	N	%
Yes	1	6.7
No	6	40.0
Unknown	6	40.0
No Answer	2	13.3

The prevalence of availability of SGM education is shown in Table 2. About 27.5% (22/80) of the medical schools provided lectures or workshops related to SGM. In addition, one school replied that they were unsure, since instructors might address this theme even though it does not appear on the syllabus.

A summary of the SGM educational coursework is shown in Table 3. About 90% of these lectures or workshops were given as required subjects. In addition, 10 medical schools provided lectures or workshops to third-year students, and nine medical schools delivered SGM curriculum to first-year students. Moreover, five of 37 schools provided SGM education in two different years (e.g., first year and third year). The median time of the lectures was 130 min (interquartile range [IQR], 135 min).

The top three course offerings with SGM lectures or workshops were Gynecology and Obstetrics, Neuropsychiatry, and Introduction to Medicine. We selected the principal lecture or workshop contents from 22 schools and presented them in Table 4. A lecture was the primary method of addressing the general concept of SGM and disorders of sexual development (DSD). One lecture approach was a class led by a person who identified with SGM groups. Another approach was a class which addressed: [1] ethical matters, such as lesbian women having a baby with artificial semination, [2] mental issues, such as anxiety and difficulties that persons with gender identity disorder (GID) and gender dysphoria, are likely to experience, and [3] sex-reassignment surgery (SRS) for GID.

The primary reasons for not offering any classes or workshops with respect to SGM were unavailability of suitable instructors or no policy regarding SGM education in the medical school. Two schools responded that SGM education is not currently necessary in medical education. In addition, in medical schools that did not provide SGM education, more than 90% responded that they did not have plans to provide this type of education, or they were unsure of their future plans (Table 5).

The top three appropriate subjects for providing SGM lectures were Gynecology and Obstetrics, Neuropsychiatry, and Urology. In addition, six schools responded that any departments could effectively provide the training within a wide range of curriculum areas. Furthermore, seven schools responded that they could not select specific subjects which could include SGM lectures. Their reasoning was that the answer depended on the purpose of the lectures.

## Discussion

The first step is to outline the structure of this discussion, with defining frameworks and elements of the core curriculum, then presenting the status of SGM education in Japan Medical Schools and limitations of the study.

**I. Van Melle framework**

- Outcome competencies
- Sequenced progression
- Tailored learning experiences
- Competency-focused instruction
- Programmatic assessment

**II. Curriculum with respect to competency classifications**

This curriculum is derived from Basic Qualities and Abilities Required of a Physician of the Model Core Curriculum [16].

- Patient care
- Knowledge for practices
- Professionalism

**III. Current status and issues facing SGM education in Japan**

- Structure of Sexual Minority Education in Japan
- Evaluation of competency-based sexual and gender minority education based on selected core model curriculum, listed above.
- Implementation
  1. Student Assessment Practices
  2. Instructional methods
  3. Tailored Learning Experiences
  4. Barriers of Dissemination in Sexual and Gender Minority Education
  5. Recommendations for disseminating sexual and gender minority education

**IV. Limitations**

In the first stages of the outline, we present the structural tools used in this study.

**I. Van Melle framework**

According to Van Melle, competency-based medical education is enhanced by five core components of CBME curricula: [i] outcome competencies, [ii] sequenced progression, [iii] tailored learning experiences, [iv] competency-focused instruction, and [v] programmatic assessment [33]. Our study addressed these elements in the following ways:

**(A) Outcome competencies**

The survey results revealed that SGM curriculum is limited in the present status of Japanese medical education. Moreover, the momentum to develop relevant SGM curriculum is also restrained.

**(B) Sequenced progression**

Our study was in too early a stage to develop a sequenced curriculum. However, our survey requested input regarding the best year(s) to present SGM material, and in which classes, which are already assigned to a sequence. Thus, in assigning material to suggested classes and academic years, an outline should begin to emerge of recommended sequenced progression for a future study.

**(C) Tailored learning experiences**

The survey responses we collected established that the most effective learning experiences associated with SGM education for medical students may be interaction with and talks by SGM individuals, themselves.

**(D) Competency-focused instruction**

Primarily, instructional methods in our survey were lectures targeting material mandated in the Medical Core Curriculum [16]. One school presented a workshop and another school held a forum in which students discussed the roles of healthcare professionals for SGM healthcare.

**(E) Programmatic assessment**

In this primary stage of the study, we did not develop a methodology for programmatic assessment, which is a learner’s progress that is continually collected, analyzed, and often supplemented with additional assessments to inform both the learner and teacher of progress towards the end of a training phase [36]. Programmatic assessment is valuable not only to the student, but to the teacher, who can evaluate whether curriculum changes have successfully promoted the competencies and outcomes in medical student training.

**II. Curriculum with respect to competency classifications**

We viewed relevant curricular content derived from the survey, utilizing the tools of *Japan’s Model Core Curriculum for Medical Education* [16] and the Van Melle framework [33] to develop recommendations for SGM competency development within the three competency classifications:

**A. Patient care**

(1) Lectures which provide background knowledge on SGM and DSD helped give students basic knowledge to elicit relevant information about sex anatomy, sex development, sexual behaviour, sexual history, sexual orientation, sexual identity, and gender identity from all patients in a developmentally appropriate manner, both sensitively and effectively. The survey revealed, however, that students were not trained to determine what information was appropriate or necessary to collect from patients.
(2) Lectures that describe the anxiety and difficulties that persons with GID and gender dysphoria are likely to experience may give students an opportunity to better perceive the mental status associated with transgender patients. Lectures which describe treatments for GID may inform students about the different alternatives in transition that transgender patients choose, which can involve one or a combination of actions, including simply altering appearance, undergoing sex reassignment surgery, or pursuing hormonal therapy, with each having its own medical implications.
(3) Lectures on DSD may help students incorporate the special healthcare needs and available options for DSD patients into their clinical training for healthcare delivery.
(4) Classes led by persons who identify with SGM groups, discussions regarding cases in which lesbian women have a baby with artificial semination, and classes which present the anxiety and difficulties that persons with GID and gender dysphoria are likely to experience may allow students to recognize the unique health risks and challenges often encountered by SGM.

**B. Knowledge for practices**

(1) Lectures which provide background knowledge on SGM may help students define and describe sex and gender differences; gender expression and gender identity; gender discordance, gender nonconformity, and gender dysphoria; and sexual orientation, sexual identity, and sexual behaviour.
(2) Lectures on DSD knowledge may aid students in understanding typical (male and female) sex development and learning the main etiologies of atypical sex development.
(3) A class in history, social problems, and discrimination regarding LGBTQ and a workgroup in which freshmen at medical school discuss news that one women’s university was considering the enrollment of a transgender student (male-to-female) may help students understand and describe underlying historical, political, institutional, and sociocultural factors in healthcare disparities experienced by the SGM populations.

**C. Professionalism**

(1) Discussion of the roles of healthcare professionals in healthcare for SGM patients provides students the opportunity to better understand relevant medical issues concerning SGM, challenges encountered, and health providers’ role in care of SGM patients.

**III. Current status and issues facing SGM education in Japan**

Four topics are essential to this study: (A) Structure of SGM education in Japan, (B) How competency-based SGM education was implemented, (C) Barriers of dissemination of SGM education, and (D) Recommendations for implementation of SGM education.

**(A) Structure of sexual minority education in Japan**

The median duration of lectures in Japan obtained in the present survey was 130 min (compared to 5 h in US and Canadian medical schools in 2010) [37]. In Japan, SGM education has only recently been initiated.

Our results revealed that SGM-related content was taught in a range of medical fields, concurring with other studies. In the above-mentioned US and Canadian study, many respondents asserted that LGBTQ-related preclinical content should be interspersed throughout the curriculum [37]. One South African study exemplified curriculum in which LGBTQ health-related content had been interspersed throughout [38]. The top three ideal subjects offering SGM education were Gynecology, Psychiatry, and Urology. On the other hand, support for offering this curriculum in a wide range of subjects was voiced, since students may associate SGM as a disorder in a specific medical area if SGM education is limited to specific clinical areas. Therefore, basic information covering topics on sexual identity, sexual orientation, gender identity, social discrimination, difficulties in gaining access to medical facilities, and judgmental care from health providers may be best taught in medical education or professionalism lectures at early stages. In addition, during clinical lectures, each subject should introduce different aspects of diseases and healthcare alongside issues by which SGM is impacted.

Regarding the health issues facing SGM, the above-mentioned US and Canadian study revealed that HIV, sexually transmitted infections (STIs), and safer sex were part of the principal LGBTQ-related topics in undergraduate medical education [37]. In the present survey, the coursework did not address HIV and sexually transmitted diseases (STDs) among men who have sex with men (MSM), and yet these diseases have been very serious public health concerns in Japan. For example, a recent resurgence in reported syphilis cases was recognized among HIV-1-infected MSM in Japan [39]. Also, incident Hepatitis C (HCV) infection is increasing among HIV-1-infected MSM non-injection drug users in Japan [40]. Lack of HIV education in SGM lectures may diminish the opportunity to educate LGBTQ patients about safer sex options, including correct and consistent use of condoms [38].

Further, if physicians recognize that MSMs are at risk of HIV infection, HIV could be detected at earlier stages and antiretroviral treatment introduced proactively [41]. Besides MSMs, transgender individuals often resist accessing Gynecology and Urology services, given that appearance and genitals may not be congruent with assigned gender [8]. As a result, their behaviour increases the risk of HIV infection [8]. Therefore, taking inclusive sexual histories and acknowledging LGBTQ sexual behaviour are crucial to making LGBTQ patients feel comfortable and ensuring that they receive appropriate preventative and diagnostic care [42]. Instructors should provide medical students with concrete knowledge of disease prevention through better comprehension of SGM health.

Regarding lecture content, Psychiatry treats anxiety and difficulties within SGM. Additionally, for surgery for sex-reassignment to be covered by insurance, the diagnosis as GID by Psychiatrists is necessary [19]. Although internationally, GID was changed to ‘gender dysphoria’ in the Diagnostic and Statistical Manual of Mental Disorders (DSM)-5 with the intention of identifying a non-psychiatric disorder, in Japan GID is still used [43]. This discontinuity may be one reason that content in one professionalism lecture touched on ‘Treatments for GID'. At survey time, few lectures touched on SRS because it was not yet covered by insurance. Moving forward, diagnosis of GID, conditions for insurance coverage, SRS, and complications of SRS should be taught in medical education. Ideally, lectures should address each group’s (e.g., Lesbian, Gay, Bisexual, Transgender, Queer, etc.) unique health issues and health needs. More specifically, education providing knowledge of unique clinical concerns and social considerations facing transgender patients, as well as building needed clinical skills, is essential to delivering compassionate, competent care [44]. A transgender patient’s health concerns should not be aggregated into LGB health issues [44]. For example, transgender individuals should have cancer screening that is based on both assigned and present gender [45].Regarding the best year of class standing to offer SGM lectures, the present study demonstrated that the first- and third-year levels were the top two. If possible, diversity-related lectures should be offered at an early stage to help students develop their own understanding before they begin the medical science content. Awosogba et al. asserted that the first 2 years of medical school were the best time to build basic core values [46]. During these 2 years, students need to acquire and demonstrate the following basic core values to excel in health services and engage in eliminating health disparities: integrity, compassion, trust, hope, humility, and respect [46]. In addition, developing an understanding of the circumstances that SGM face may strongly influence students’ clinical development [47]. After students learn basic core values in early years, they also need to practice working with SGM patients appropriately at or even before clinical clerkship begins.

(**B) Evaluation of competency-based sexual and gender minority education**

Japan’s competency-based core model stipulates not only the ability of explaining gender formation and sexual orientation, but also the ability of explaining gender identification ‘considerations’ [16]. This competency applies not only to the ability to elucidate conditions or terminologies for SGM, but also gender identification of patients. Therefore, **the overall goal** of this education is that physicians learn to treat SGM patients as effectively as heterosexual and cisgender patients [20], and based on this goal, we examined identified *reforms* in designing SGM curricular competencies (Table 6). By and large, present Japanese outcome competencies are underdeveloped. As a result, two essential measures are: (1) a significant shift in behaviours within Japan’s medical profession and (2) enhanced medical knowledge and clinical training with respect to SGM patient concerns.

**Table 6.** Identified reforms in designing sexual and gender minority curricular competencies in Japan

Role of **Competencies**	Reforms
Ability to explain gender formation, sexual orientation, and ways of consideration for gender identification **Patient Care**: Sensitively and effectively eliciting relevant information about sex anatomy, sex development, sexual behaviour, sexual history, sexual orientation, sexual identity, and gender identity from all patients in a developmentally appropriate manner. Describing the special health care needs and available options for quality care for transgender patients and for patients born with DSD Recognizing the unique health risks and challenges often encountered by the individuals described above. **Knowledge for Practice** Defining and describing the differences among: sex and gender; gender expression and gender identity; gender discordance, gender nonconformity, and gender dysphoria; and sexual orientation, sexual identity, and sexual behaviour Understanding typical (male and female) sex development and knowing the main etiologies of atypical sex development Understanding and describing historical, political, institutional, and sociocultural factors that may underlie healthcare disparities experienced by the populations described above. **Professionalism** Accepting shared responsibility for eliminating disparities, over bias Developing policies and procedures that respect all patients’ rights to self-determination	i) List open-ended questions that can be used with any patient to elicit a sensitive and accurate history ii) Simulation exercise with peers or simulated patients demonstrating how to obtain a patient history of sexual behavior, sexual history, sexual orientation, and gender identity iii) Clinical practices such as how to ask patients’ their sexual orientation and gender identity in clinical settings i) Simulation with peers or simulated patients to elicit tailored health messages of sexual and gender minorities i) Problem Based Learning regarding sexual minority patients
**Implementation** Student Assessment Practices Instructional methods Tailored Learning Experiences	Multiple-choice exams Open-ended questions Multiple assessment tools i) Providing evaluation of the educational process with student responses to surveys. ii) Providing a student with feedback as a result of programmatic assessment. Small group discussion Small group discussion
**Overall goals**	To ensure that physicians can treat sexual and gender minority patients as effectively as heterosexual and cisgender patients.

**(1) Patient care**

Existing teaching methods were almost entirely didactic lectures for students to increase their knowledge regarding SGM, although talks by SGM themselves might help students to understand the unique heath risks and challenges from their authentic experience. Therefore, to achieve the overall goal, not only lectures, but also clinical practice, are important. How to ask a patient’s sexual orientation and gender identity should be taught in preclinical settings, as patient answers are very important when initiating clinical clerkship [21].

 However, since students and patients are not currently familiarized with these questions in Japan, at the beginning of the exercise, i) students should list open-ended questions that can be used with any patient to elicit a sensitive and accurate history, and ii) rather than engaging patients, exercises with peers and simulated patients may be a more comfortable, much safer, and more effective approach for students [35].

**(2) Knowledge for practice**

Lectures which provide background knowledge on sexual and gender minorities and DSD are appropriate for students to increase knowledge for practice. However, to understand and describe historical, political, institutional, and sociocultural factors that underlie healthcare disparities experienced by the populations described above, group work discussing relevant factors in addition to simulation training with peers or simulated patients to elicit tailored health messages of SGM is important [35].

**(3) Professionalism**

(i) To accept shared responsibility for eliminating disparities and bias, and ii) develop policies and procedures that respect all patients’ rights to self-determination [35], consideration in preclinical training should be given to integrating SGM concerns into a problem-based learning curriculum, in addition to the discussion the roles of healthcare professionals in SGM patients [48].

**C. Implementation**

Considerations for elements of implementation in the context of competency-based medical education take the following shape:

**(1) Student assessment practices**

Although not requested of participants, assessment practices focusing on promoting student growth and development are important [33]. Dogra et al. claimed that medical schools must always consider a range of teaching and assessment strategies, including reflection in practice to examine student knowledge and level of comprehension [49]. Especially if the purpose of lectures was to increase students’ knowledge of SGM, assessment of learner acquisition of new knowledge is crucial [35]. Multiple-choice exams before and after class are suitable options, along with open-ended questions after class to describe or assess special or unique needs of SGM patients [35]. Additionally, in the future when students begin clinical practice, multiple assessment methods are more appropriate for assessing students’ behaviour [35].

**(2) Instructional methods**

Primarily, instructional methods in our survey were lectures, and only one school provided a workgroup in which students discussed sexual minority issues. However, feedback processes are an essential part of instruction for quality improvement of education [50]. For example, one medical school in London utilized feedback to assess the initial lecture regarding sexual orientation, and for the following lecture, they succeeded in expanding the program based on the feedback from students [50]. Feedback takes many forms, providing an evaluation of the educational process with student responses to surveys, or providing a student with feedback as a result of programmatic assessments [33,51]. In medical education, feedback must be an integration of the learning process, as it encourages the students’ knowledge and skills improvement and enhances their professional development [51].

**(3) Tailored learning experiences**

A talk led by SGM, themselves, may encourage medical students to learn more about health disparities of SGM [21]. As an example, one research project focusing on general college students revealed that those in direct contact with LGBTQ individuals more significantly understood them than students without such experience [6]. This result can also be applied to medical students. Therefore, more interpersonal interactions, such as small group discussions in which an SGM individual leads the students, may contribute to a positive change in student attitudes [21].

**(4) Barriers of dissemination in sexual and gender minority education**

The rate of prevalence of SGM medical education in Japan was only 28%. This percentage was much lower than that of US and Canadian medical schools (70%) in 2010 [37]. Primarily, two obstacles existed for disseminating this education in Japan. The first was incomprehension and the second was infeasibility of this education, even if faculties understood its necessity.

 Evidence of incomprehension came from: (1) the low response rate (46%) (e.g., 85% in United States and Canada) [37]: (2) unconstructive answers: two schools answers indicated they did not want to participate in our survey; and (3) answers which denied the necessity of this education: two schools stated that they did not need an SGM curriculum at present. Incomprehension may arise due to the antiquated mindsets of faculty members and stigma toward SGM. Although one survey stated that younger people more easily accept SGM individuals [52], about 40% of college students in 2015 voiced nonacceptance of LGBTQ individuals [6]. Thus, it is no wonder that faculty members of Japanese medical schools, who are older than university students, are hesitant to engage in SGM outreach and research to provide a meaningful curriculum. Although recent data remain scarce, one study conducted in 2005 focusing on gay and bisexual men showed that in general school curriculum, 79% of the respondents did not learn about gay males at all, 4% learned that being a gay male was abnormal, and 11% learned negative information about gay men [4]. Therefore, the generation of faculty members of a majority of current medical schools is thought to have never studied SGM issues. Also, as for stigma, Japanese people generally have a strong bias towards SGM and involvement in the activities supporting sexual minorities [11], which might help account for the low response rate to our survey. Sexual-and-gender-minority-related issues might have discouraged participation in the present survey.

 Regarding the perceived infeasibility of this education, two reasons are ‘no suitable instructors’ followed by ‘no policy in the medical school'. On the other hand, among the strategies cited as currently or potentially successful in increasing LGBTQ-related content in the curricula of US and Canadian medical schools, using ‘Curricular materials focusing on LGBTQ-related health/health disparities’ was the most popular strategy [37]. The second most popular strategy was identifying ‘Faculty willing and able to teach LGBTQ-related curricular content’ [37]. In Japan at present, cultivation of human resources is the first priority for integrating inclusive content regarding LGBTQ individuals into the medical course curriculum. The second priority is developing school policy for SGM education. However, as above mentioned, the faculties in most medical schools are too antiquated to consider the school policy of SGM education.

**(5) Recommendations for disseminating sexual and gender minority education**

**Figure 1.**
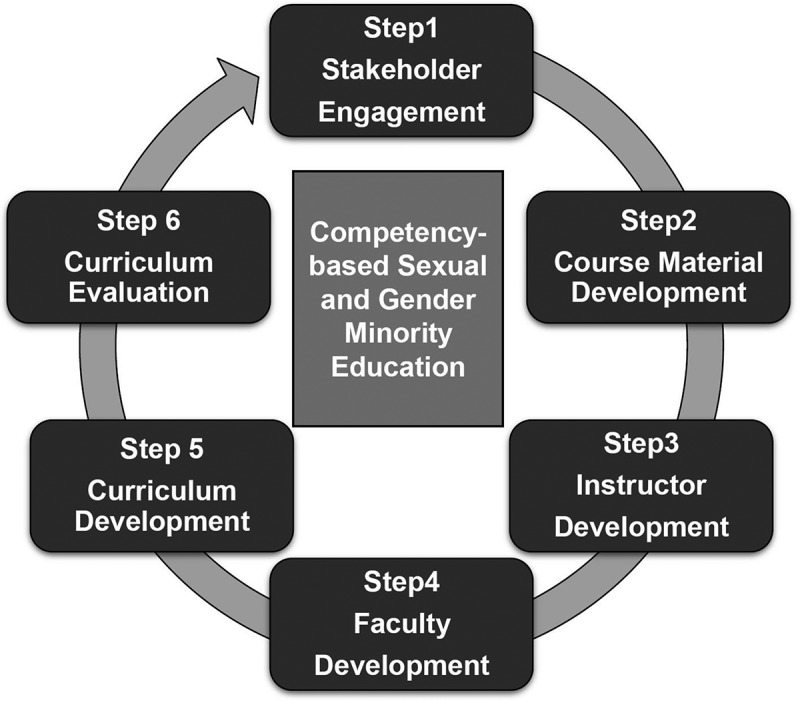
Monitoring framework for developing competency-based sexual and gender minority education in Japanese Medical Schools

When we incorporate SGM education into the present medical curriculum, the following steps are recommended. These steps are explained within the framework (Figure 1) based on the program evaluation of Utah State University [53]. *The first* step is engaging appropriate stakeholders (i.e., government, medical educators and students, members of LGBTQ community). *The second* step is developing a textbook or an educational guide for SGM education. Medical schools which currently provide these programs could be identified, offering a path to designating appropriate instructional candidates. Subsequently, with government support, these experts would create course materials which best fit Japanese medical settings through focus groups. *The third* step is developing a diverse curriculum team for each medical school, which should include passionate instructors, representatives of SGM in the medical community, administrators, and other positions. The expert instructors identified in our present survey would also help train instructors of other medical schools not currently offering substantial coursework covering SGM. *The fourth* step is conducting faculty development, with faculty members encouraged to become acquainted with members of the SGM groups directly. This opportunity may help faculty members develop more positive impressions of SGM [21] in relating to their humanity. *The fifth* step is curriculum development, in which expert instructors could help medical schools integrate SGM training congruous with current course offerings at each university, pinpointing the most advantageous subject areas and class levels. Developing an SGM curriculum in each medical school within a framework is an effective approach to establishing school policy SGM education. Implementing outcome-based standards in SGM medical education throughout Japan is important, as derived from the experiences and knowledge accrued by a growing base of expert instructors. Also, the framework and educational materials should be specific to Japan, as the culture and history of SGM in Japan can vary substantially from those of other countries. The *last* step is curriculum evaluation, for which the Japanese government should be provided a reliable and tested schema of working policies and a detailed framework developed from successful curricula. With these data, new national policies can be promulgated.

**IV. Limitations**

This study has several limitations. *First*, only 46% of the medical schools responded to this questionnaire survey. Therefore, the status of SGM education in medical schools not responding is unknown. It may be possible that only medical schools interested in this matter responded. *Second*, survey respondents may not have been fully cognizant of all available lectures provided in their institutions. Therefore, it is possible that several instructors included SGM health in their lectures, but the content was not reflected in the syllabus. Third, we would have been best served asking respondents to share their own strategies for presenting SGM lectures in an environment where acceptance is still limited. Fourth, this survey was not a quantitative survey, and therefore, we did not request the details of lectures or workshops. An example for improvement would be to ask what aspects the instructor emphasized in SGM lectures and required competencies of each lecture. Therefore, as a next step, we should conduct qualitative surveys, i.e., interview surveys, to explore the details of SGM education in Japan.

## Conclusions

We concluded that only 22 medical schools in Japan provide any kind of SGM education. In addition, the overall content of the present SGM education may already target certain competencies. However, education should adhere to competencies more strictly. Promoting clinical practices that treat SGM patients with the same effectiveness and compassion as heterosexual and cisgender patients should be a critical element of content. Lectures should reference STDs among SGM, since physicians with this knowledge can prevent STD epidemics among SGM, as well as the spread to the general public. Special health needs of transgender patients should be taught in lectures, as SRS begins to be covered by insurance. In the future moreover, lectures should deal with each groups’ health challenges and tailored needs. Last, we found an unwillingness among some faculty or schools to provide this education effectively, and among others, a lack of preparation and knowledge to implement this education, including no suitable instructors and no formal school policy. Therefore, to disseminate competency-based SGM education in Japanese medical schools, six steps are suggested, using a monitoring framework: (1) engage government, (2) develop course material, (3) develop instructors, (4) conduct faculty development with opportunity to interact with sexual minority individuals, (5) create curriculum through the authority of developing school policy for this education, and (6) evaluate effectiveness of curriculum through outcomes with government oversight.

## Appendix

### Survey of Sexual and Gender Minorities Education in Medical Schools

Note: In this survey, the term ‘sexual and gender minorities’ is used to refer collectively to persons who self-identify their sexual orientation and gender identity (SOGI) within the lesbian, gay, bisexual, transgender, or queer/questioning (LGBTQ) spectrum, and to persons with a disorder of sex differentiation (DSD), a state of having atypical development of the genitalia in relation to the chromosomes or gonads.

Q1. At your institution, do you currently offer any class (or workshop) regarding sexual and gender minorities within the 6-year medical program? (Please encircle your answer here and in the questions that follow.)

1) Yes→ Go to Q2-1

2) No→ Go to Q3-1

3) Unsure→ Go to Q3-2

If you answered ‘Yes’ in Q1, please answer the following questions:

Q2-1. Is that class (or workshop) required or elective? (multiple answers allowed)

1) Required2) Elective3) Unsure

Q2-2. To which year of students is that class (or workshop) offered? (multiple answers allowed)

1) 1^st^ year2) 2^nd^ year3) 3^rd^ year4) 4^th^ year5) 5^th^ year6) 6^th^ year7) Unsure

Q2-3. If possible, please provide details about the class (or workshop) including time allocation, class title, and content summary.

**Table:**

	Academic Level	Course title that includes the class (or workshop) in its syllabus	Time allocation	Contents (summary)
*e.g.*	*4^th^ year*	*Urology*	*Part of a 90-min class*	*About androgen insensitivity syndrome*
*e.g.*	*5^th^ year*	*During clinical training in Psychiatry*	*Unsure*	*About LGBTQ Issues*

If you answered ‘No’ in Q1, please answer the following questions:

Q3-1. What is the reason (*or* are the reasons) for not offering any class (or workshop) about sexual and gender minorities? Please encircle all appropriate answers. (Multiple answers allowed)

1) Do not have concrete ideas about what to do

2) Curriculum is too tight to offer a class

3) No suitable instructors

4) Unable to find standard teaching materials

5) University has no policy about sexual and gender minority education

6) Currently no need has been identified

7) Others

If you answered ‘No’ or ‘Unsure’ in Q1, please answer the following questions:

Q3-2. Do you have any plans to offer a class (or workshop) about sexual and gender minorities?

1) Yes, have plans2) No plans3) Unsure

We would like to ask all respondents to answer the following question:

Q4. When offering classes (or workshops) about sexual and gender minorities in medical schools, which medical courses or fields do you think are the most appropriate to host these classes (or workshops)?
